# Embryonic ethanol exposure on zebrafish early development

**DOI:** 10.1002/brb3.2062

**Published:** 2021-05-03

**Authors:** Jaquelinne Pinheiro‐da‐Silva, Ana Carolina Luchiari

**Affiliations:** ^1^ Physiology and Behavior Department Federal University of Rio Grande do Norte Natal Brazil

**Keywords:** alcohol, embryogenesis, morphology

## Abstract

**Introduction:**

Embryonic exposure to ethanol leads to a condition of physical, behavioral, and cognitive deficiencies named fetal alcohol spectrum disorders (FASD). The most severe variations are in fetal alcohol syndrome (FAS), which is easier to diagnose and not studied in animal models. On the other side, the pFAS (partial fetal alcohol syndrome) includes cases of alcohol‐related congenital disabilities and neurodevelopmental disorder with an inconclusive diagnosis. In recent years, the zebrafish has become a valuable model to study FASD and its variations.

**Methods:**

This study characterizes the zebrafish embryonic and larval development after low and moderate ethanol concentration exposure. Fish eggs were exposed to 0.0%, 0.25%, 0.5%, and 1.0% ethanol at 24 hr postfertilization, and embryonic development was observed every 8 hr up to 120 hpf. It evaluated movements, phenotypic abnormalities, hatching, cardiac function and heartbeat frequency, larvae length at 120 hpf, and the apoptotic cells' fluorescence stained with acridine orange.

**Results:**

Embryonic exposure to 0.5% and 1% ethanol presented reduced body size, decreased heartbeat rate, higher numbers of apoptotic cells, and hatching time differences.

**Conclusions:**

Our results suggest any ethanol exposure during embryogenesis can be harmful and reinforces zebrafish as a suitable model for fetal alcohol spectrum disorders (FASD).

## Graphical abstract

The alcohol intake by pregnant women is a worldwide health concern. Zebrafish has become a valuable model to study fetal alcohol spectrum disorders. Even low doses of ethanol exposure during embryogenesis could affect zebrafish development and induce apoptosis.

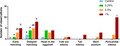

## 1 INTRODUCTION

Alcohol (ethanol) is a licit drug in which consumption is well accepted and stimulated worldwide; however, the impact on society's health is worse than those of illicit drugs. One of the most common problems associated with alcohol is the intake of pregnant women. It can lead to fetal alcohol spectrum disorders (FASD), an umbrella term used to categorize the numerous diagnostic outcomes of prenatal alcohol exposure (Barr & Streissguth, 2001; Roozen et al., 2016). This condition covers physical, behavioral, and cognitive deficiencies, and presents few clinic variations: the most known, fetal alcohol syndrome (FAS), that is, the severe spectrum, characterized by the full phenotype of neuropsychiatric impairment and severe congenital disabilities (Guerri et al., 2009; Jones et al., 1973), and the partial fetal alcohol syndrome (pFAS) (Jones & Smith, 1973; Kingdon et al., 2016) that includes alcohol‐related birth defects (ARBD), the nondysmorphic type with a neurobehavioral disorder, and alcohol‐related neurodevelopmental disorder (ARND), the brain damage type with impairment in functional behavior (Hagan et al., 2016; Hoyme et al., 2005). Both ARBD and ARND do not meet all the criteria used for diagnosis, and the increased interindividual variations turn the diagnosis inconclusive (Hagan et al., 2016).

It is estimated that one in every 13 early alcohol‐exposed infants will show FASD (Lange et al., 2018), and around 25% of individuals with FASD fit the intellectual disability criteria (Streissguth, 1997). Nevertheless, alcohol early exposure affects development depending on the embryo's age and pattern of exposure (Lange et al., 2018). Many studies have been trying to identify the mechanisms of action of this drug at all levels, and due to the obvious limitation of human studies, most of the research on FASD currently uses translational animal models. Rodents are an important model and the most applied in alcohol‐related studies (Gil‐Mohapel et al., 2019). However, fetal development inside the uterus makes it challenging to analyze alcohol teratogenic effects. Besides that, drug exposure and evaluation of alcohol levels are invasive and may cause stress to the mother interfering with the results. The drug concentration and length of exposure resulting in specific phenotypes are harder to be determined in mammals since the mother's metabolic functions must also be considered and drug effects can only be seen after birth. On the other hand, the zebrafish (*Danio rerio*) that was first brought to the laboratories to assist in developmental biology (Grunwald & Eisen, 2002) appeared as a promising alternative to fetal ethanol exposure studies.

As a fish, this small vertebrate has some practical advantages, in addition to its small size, high fertility, and short development time (Stewart et al., 2014). They present external fertilization; thus, controlling ethanol concentration and exposure time is more manageable and does not imply parental manipulation. The eggs are transparent, so phenotypic alterations in specific embryonic stages are readily determined without development interference. Alcohol easily crosses the chorion, facilitating further studies during embryonic development (Blader & Strähle, 1998). Moreover, the fast development of zebrafish (3 months until adult stage) allows the comparative study of few generations in a time interval significantly shorter than other animal models (Kalueff et al., 2014), making it possible to investigate the effects of alcohol on the following generations, in addition to various combinations of “concentration × length × developmental period.”

Zebrafish embryos exposed to ethanol show growth deficiency at pre‐ and posthatching, and phenotypic abnormalities similar to FAS children, suggesting that the ethanol exposure harms the same molecular mechanism in both humans and zebrafish (Arenzana et al., 2006; Bilotta et al., 2002; Carvan et al., 2004; Chmielewski et al., 1997; Matsui et al., 2006; Tenkova et al., 2003). Although recent studies have explored the zebrafish as an animal model to ethanol exposure during development, this research focuses on a high concentration of alcohol and embryonic malformations most related to FAS. At the same time, there is still a gap in the knowledge of possible impairments after lower concentrations of ethanol exposure.

The zebrafish brain development occurs on the late segmentation stage until the pharyngula phase (around 24 hr postfertilization—hpf), equivalent to the early gestational period in humans, which is the most critical phase of embryogenesis due to the high level of neuronal cell birth. For instance, studies inducing embryonic stress at this time point resulted in numerous apoptotic cells (Hashimoto et al., 1998; Yamashita, 2003). Despite the studies' significant progress on ethanol effects on embryonic development, results are still contradictory and inconclusive (Pan et al., 2012). Therefore, this study aimed at characterizing the zebrafish embryonic development after low and moderate ethanol concentration exposure at the 24 hpf developmental phase. With these results, we contribute to a better view of the available data using different zebrafish strains and ethanol concentration, as well as suggest specific features that could contribute to the diagnosis of pFAS condition.

## 2 METHODS AND MATERIALS

### 2.1 Embryonic collection

Adult zebrafish (*Danio rerio*), 6 months, in‐house reared, wild‐type (WT) strain, were used for this study. For breeding, fish were set up in four different breeding tanks (2 males: 1 female/tank), with only visual and chemical contact (fish separated by a partition) and let overnight. The partition was removed on the following morning's first light hour and fish could mate for 1 hr. This procedure ensured that we knew the exact window time of fertilization. Then, eggs from the four breeding tanks were collected and placed in a plastic tray with system water until 24 hr postfertilization (hpf). The photoperiod was kept at 12‐hr light: 12‐hr dark. All experimental protocols were approved by the Committee for Animal Use of Federal University of Rio Grande do Norte (CEUA 004002/2017).

### 2.2 Embryonic exposures and developmental analyses

At 24 hpf, embryos were randomly chosen and transferred to 24‐well multiwell plates, 6 embryos per well, and exposed to four different ethanol concentrations (0%, 0.25%, 0.5%, and 1%) for 2 hr. These concentrations were chosen based on previous reports that managed to measure the alcohol concentration inside the zebrafish eggs after 2 hr of exposure, showing that about 1/25 and 1/30 of the external alcohol concentration reach inside the egg (Fernandes & Gerlai, 2009). Additionally, these concentrations do not cause gross morphological deformities (Buske & Gerlai, 2011). After 2 hr of ethanol exposure, the eggs were washed twice, and embryos were transferred to clear multiwell plates (6 embryos/well) and raised to 120 hpf in the same standard conditions described above.

To analyze zebrafish embryonic development till the larval stage, we established endpoints every 8 hr; thus, the analysis took place three times a day to cover all gaps and spot the main differences for a full developmental window. Observations were made at 8, 16, 24, 32, 40, 48, 56, 64, 72, 80, 88, 96, 104, 112, and 120 hpf. All developmental analyses were performed in an isolated room, maintained at ~28°C.

A total number of 60 eggs were used for each ethanol concentration exposure (10 replicates, with 6 larvae each). Dead embryos were counted and removed during every observation to avoid contamination. Developmental changes were strictly observed using a binocular stereoscopic microscope. Spontaneous movements, phenotypic abnormalities, and hatching were counted at each time point. Ten embryos from each treatment were recorded for 1 min for cardiac function evaluation, and the rate of the heartbeats was calculated. Measurements of embryonic length were done only at 120 hpf, using an adapted micrometer. The distinction between normal and abnormal development was established using zebrafish embryogenic description by Kimmel et al. (1995).

### 2.3 Apoptotic cells quantification

Acridine orange staining was performed to investigate cellular apoptosis in ethanol exposed embryos, following the protocol by Kim et al. (2014). Acridine orange can permeate apoptotic cells and binds to DNA, whereas healthy cells are nonpermeable to acridine orange. Thus, it stains necrotic or late apoptotic cells. Five embryos from each treatment were exposed to ethanol at 24 hpf, for 2 hr. After that, animals were washed twice, transferred to 96‐well plates, and treated with acridine orange solution (7 μg/ml) for 1 hr, at 28 ± 1°C in a dark room. Next, embryos were washed twice and anesthetized by ice before observation under the fluorescence microscope (Nikon CFI60, Eclipse Ti). Animals were photographed on the same settings to standardize the background: DSQi1Mc 12 bit, auto‐exposure 10 s, and analog gain of 16.0×. For each larva, the fluorescence intensity was quantified using the ImageJ software 1.52p (National Institutes of Health, USA).

### 2.4 Statistical analyses

Data were analyzed for outliers and tested by the Shapiro–Wilk normality test. The Kaplan–Meier curve was built for percent survival analysis, and a comparison was performed using the chi‐square test and the log‐rank test. Given the non‐normal data distribution, total body length, and heart rate between treatments were compared by Kruskal–Wallis and Friedman test, respectively, both followed by Dunn's post hoc test. Two‐way ANOVA was used to test the between‐subject main effect of ethanol concentration versus phenotypic observations, followed by Fisher's LSD post hoc test. The relative fluorescence intensity and the number of acridine orange stained cells were compared for each ethanol treatment using Kruskal–Wallis, followed by Dunn's post hoc test. GraphPad Prism 7.0 (GraphPad, La Jolla, CA) was used for all statistical analyses, and the significance level was set to 0.05.

## 3 RESULTS

**FIGURE 1:**
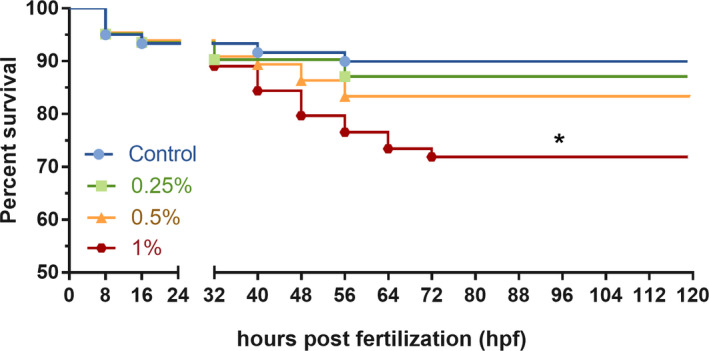
Percent survival curves for the Eth 0.25%, Eth 0.5%, Eth 1%, and control group. The gap between the axis illustrates the time point the groups received ethanol treatment (24 hpf), symbols in each line represents the time point with death registered for each group. (*) means statistical significance, *p* < .05, Kaplan–Meier curve followed by log‐rank test, *n* = 60/group

The log‐rank test was used for the trend to determine whether the difference between survival curves was more than expected by chance. Although all ethanol treatments applied presented more than 50% of survival, data from Eth 1% were statistically lower than the other groups (log‐rank: χ^2^ = 4.82, *p* = .03). The survival rate of the embryos exposed to ethanol was 90% for the control group, 87% for Eth 0.25%, 83.33% for Eth 0.5%, and 71.8% for Eth 1%. (see Figure 1 for details). The first death was registered at 32 hpf and the last death at 72 hpf.

Our results show that embryos treated with ethanol presented modest morphological defects such as heart, tail, and yolk sac edema, and curved posture. Ethanol exposure also caused a hatching delay. Most of the abnormalities were found for Eth 1% group and minor effects for Eth 0.5% (Table 1). Images of the morphological abnormalities observed can be found in Supporting Information S1.

**TABLE 1:** Summary of phenotypes detected in zebrafish embryos exposed to 0.25%, 0.5%, and 1% ethanol at 24 hpf, observations were made every 8 hr until the larval stage

Time (hpf)	Phenotype	Control (0%)	Eth 0.25%	Eth 0.5%	Eth 1%
8	Bud stage Visible somites	N Beginning	–	–	–
16	Spine shape Tail separating	N N	–	–	–
24	Eye formation Head separating Visible notochord Body movement Tail free Hatching	N N Low N – –	N N Low N – –	N N N N – –	N N N N Y Premature
32	Heartbeats Body movement Pigmentation Body reflexes Hatching	Y N N N –	Y N N N Y	Y Low N Low Y	Y Low N Low Y
40	Body mobility Abnormality	Low –	Low –	Low Yolk sac anomaly	Low Curved posture/pericardial edema
48	Heart shape	N	N	Pericardial edema	Heart edema
56	Tail shape Abnormality	N –	N –	Bent –	Bent –
64	Pectoral fin Abnormality	N –	N –	N –	N Delayed pigmentation
72	Swimming bladder Mandible Abnormality	N N –	N N –	N N Head in the eggshell	Minor enlargement N Head in the eggshell
80	Eye movement Startle response	N N	N N	N N	N N
88	Hatching Abnormality	Total –	Total –	Minor delayed/pericardial edema	Minor delayed –
96	Balanced posture Thigmotaxis	Mostly Mostly	Mostly Minor delayed	Mostly Minor delayed	Mostly Minor delayed
104	Body shape	N	N	N	Bent
112	Yolk sac Abnormality	Mostly absorbed –	Mostly absorbed –	Minor delayed –	Minor delayed Tail edema/heart edema
120	Body shape Swimming	N Y	N Y	N Y	Bent Y

Note

N: normal, Y: yes, (–) any register.

**FIGURE 2:**
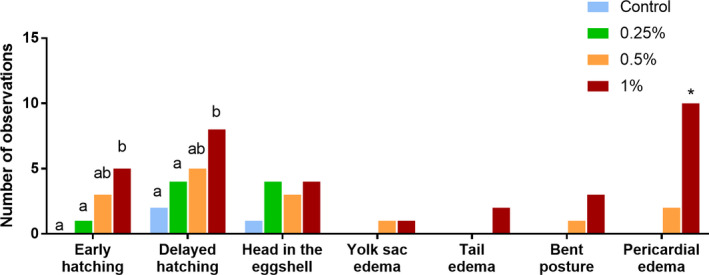
Number of phenotypic abnormalities episodes observed during 120 hr of zebrafish development, for all ethanol treatments + control. Early hatching was considered before 40 hpf, and delayed hatching was considered after 72 hpf (see supplementary material for images of morphological abnormalities). Values are mean ± *SEM*. (*) and different letters indicate statistical significance among groups, *p* < .05, two‐way ANOVA followed by Fisher's LSD test, *n* = 60/group

The observed characteristic features related to ethanol effects were compared between treatments and are shown in Figure 2. Two‐way ANOVA revealed statistical significant effect of ethanol concentration (*F*(3, 252) = 20.57, *p* < .0001) and phenotypic observations (*F*(6, 252) = 8.56, *p* < .0001), and also statistical significance for interaction terms (*F*(18, 252) = 2.16, *p* = .004).

Early hatching was considered for the larvae that hatched before 40 hpf, the time point at which the control group started to hatch. Delayed hatching was considered for hatches occurring after 72 hpf, a time point in which >70% of control animals have hatched. Fisher's LSD analysis revealed statistical significance in hatching time between Eth 1% compared with control (*p* = .001) and Eth 0.25% (*p* = .009). Besides early or delayed hatching, several larvae were observed hatched with the head remaining in the eggshell at around 72 hpf (Supporting Information S1), resulting in the larva not being counted as total hatched. As this occurrence presented no statistical difference between groups, we assume that this was not related to exposure to the drug exposure. Moreover, yolk sac edema, tail edema, and bent posture were observed for Eth 0.5% and Eth 1% groups, even though no statistical significance was found for these phenotypes. However, the exposure to Eth 1% caused significantly more pericardial edema than other treatments (*p* < .01).

**FIGURE 3:**
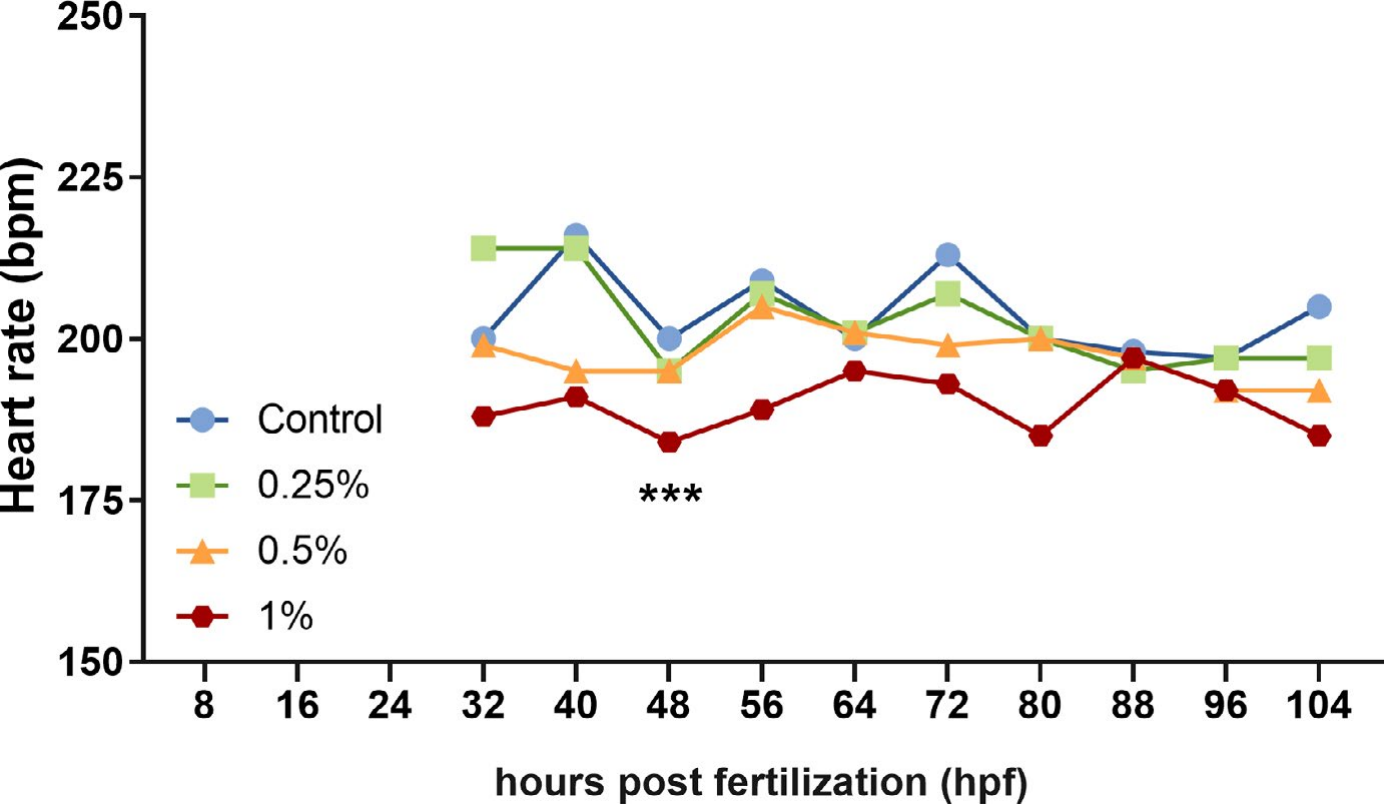
Line graph showing heart rates in beats per minute (bpm) counted for embryos treated at 24 hpf, with Eth 0.25%, Eth 0.5%, Eth 1%, and control group. Data show rates since the first heartbeats detected until the last time point in which they could be seen through the embryonic transparency (from 32 hpf to 104 hpf). (*) means statistical significance, *p* < .05, Friedman's test followed by Dunn's test, *n* = 10/group

Figure 3 shows data of heartbeats counting from 32 hpf (time point in which the heart starts to beat) to 104 hpf (after this point animal presented full pigmentation and the heartbeats counting could not be accurate). Friedman's ANOVA test showed statistical significance in heart rates between the ethanol treatments (*F* = 20.01, *p* = .0002). Dunn's post hoc test indicated that embryos treated with Eth 1% presented lower heartbeat rates than the control group (*p* < .01) and Eth 0.25% (*p* < .01), especially after 80 hpf.

**FIGURE 4:**
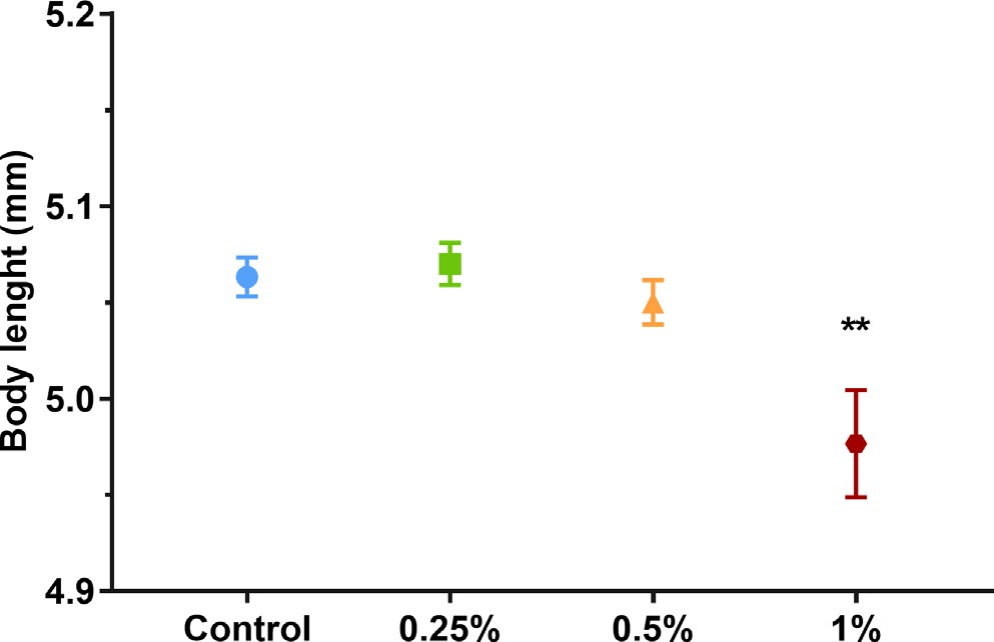
Measures of body length + *SEM* for Eth 0.25%, Eth 0.5%, Eth 1%, and control group, at 120 hpf. Animals were randomly selected for each group. (**) represent statistical significance, *p* < .05, Kruskal–Wallis followed by Dunn's test, *n* = 30/group

Body length was registered and compared between treatments when embryos reached 120 hpf. Kruskal–Wallis analysis showed statistical significance between groups (*H* = 13.82, *p* = .003; Figure 4). Dunn's post hoc test showed that Eth 1% group presented a growth deficit compared with the control (*p* = .002).

**FIGURE 5:**
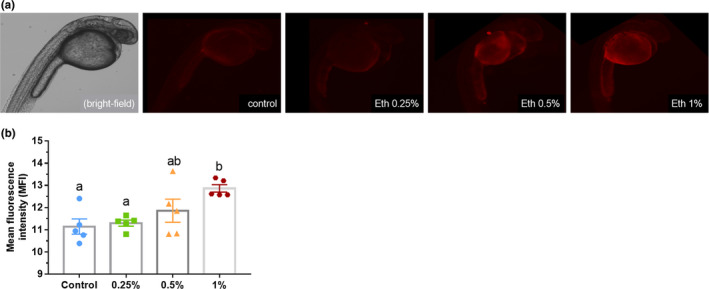
(a) Evaluation of apoptosis in zebrafish larvae exposed to Eth 0.25%, Eth 0.5%, Eth 1%, and control group, and treated with acridine orange solution. A bright‐field image of a 24 hpf embryo is shown for comparison. Note the increase in fluorescence in Eth 0.5%‐ and Eth 1%‐treated animals compared with the control. (b) Graphical representation of mean + *SEM* of mean fluorescence intensity, calculated by ImageJ. Different letters represent statistical significance between groups, *p* < .05, Kruskal–Wallis followed by Dunn's test, *n* = 5/group. Scale bar: 1 µm

Quantification of fluorescence intensity shows that the apoptosis degree increased according to the ethanol concentration applied (Kruskal–Wallis *H* = 9.70, *p* = .02; Figure 5). No significant change was found between lower concentrations, but Eth 1% differed significantly from the control group (*p* = .003) and Eth 0.25% (*p* = .018).

## 4 DISCUSSION

We observed that ethanol concentrations that are not usually considered teratogenic could affect zebrafish embryonic development. The embryonic exposure to 0.5% and 1% ethanol displayed reduced body size, decreased heartbeat rate, higher numbers of apoptotic cells, and hatching time differences. The reduced ethanol concentrations used here could not lead to a condition called fetal alcohol syndrome (FAS), but it contributes to developing issues that may later affect behavior and cognition. The characterization of all embryonic developmental stages to the early larval phase after three reduced ethanol concentration indicates that any ethanol exposure can be harmful during embryogenesis and reinforces zebrafish as a suitable model for fetal alcohol spectrum disorders (FASD).

The FASD continuum is much more prevalent than the other forms of the disorder, like fetal alcohol syndrome (FAS) (May et al., 2014; Sampson et al., 1997). However, due to huge variation caused by ethanol concentration, the period of exposure, and individual profile, translational animal models as the zebrafish gained momentum and have been helping scientists recreate impairments observed in human embryonic ethanol exposure (Fernandes et al., 2019; Marrs et al., 2010; Muralidharan et al., 2015; Shan et al., 2015). The FASD continuum leads to a range of symptoms from mild behavioral changes to more severe cognitive and social impairment, and the borders between FASD and FAS are challenging to be established as it depends on several factors including individual variations in response to ethanol (Terasaki et al., 2016). While other authors revealed higher mortality and several morphological defects caused by higher ethanol concentrations (1.5%–3%), which lead to the conditions considered FAS (Bilotta et al., 2004; Blader & Strähle, 1998; Sylvain et al., 2010), the effects and survival rate observed for 1% ethanol (~70%) makes it questionable if this concentration is indeed moderate.

Several studies on zebrafish development after ethanol exposure use relatively high ethanol concentrations and long duration of exposure. For instance, Carvan et al. (2004) used ethanol concentrations from 0.02% to 2.00% at 4 hr postfertilization (hpf) for a prolonged period. Bilotta et al. (2004) exposed 0 to 48 hpf to 1.5% or 2.9% ethanol during 8 hr. Arenzana et al. (2006) treated 4.7 hpf embryos with 1.5% or 2.4% ethanol for 20 hr. All these studies show high mortality and morphological changes consistent with FAS.

In the present study, we have not found any apparent morphological changes, such as eye or head reduced size, yet ethanol concentration was low, and the period of exposure corresponded to only 2 hr at 24 hpf. However, previous research in which zebrafish were exposed to Eth 1% and Eth 3% during the same time point we applied here could detect behavioral effects at concentrations that did not cause malformations (Bailey et al., 2015). In this study, the chosen methodology was thought to resemble moderate drinking during the first trimester of pregnancy, the period that many mothers are not aware of their state. Thus, the reduced ethanol concentration (0.25%–1%) is not expected to cause structural deformities to the embryo. However, even being low, these concentrations can be responsible for some level of toxicity that affects brain development and leads to functional disruption resulting in behavioral problems. Briefly, Baggio et al. (2020) found that 0.5% and 1% ethanol reduced Na+‐dependent glutamate uptake, reduced glutamate binding to brain membranes, and decreased Na+/K+ ATPase activity in young adult zebrafish. These changes could be implicated in the increased anxiety‐like behaviors observed as early as in the fry stage (Pinheiro‐da‐Silva et al., 2020) and maintained to adulthood in the zebrafish FASD model (Baggio et al., 2018). Therefore, as it has been shown that even short embryonic ethanol exposure induces brain and behavioral anomalies that mimic some aspects of the behavioral changes observed in FASD children, we detailed the effects of lower ethanol concentrations and for a shorter period in zebrafish embryo to the larval stage.

Ethanol exposure was observed to affect hatching time (Figure 2; Table 1). The mechanism of hatching is a combination of biochemical and behavioral processes. While the high choriolytic enzyme (HCE) and low choriolytic enzyme (LCE), synthesized by the chorion digestion gland, are sensitizing the protective layer of the egg, the animal performs spontaneous movements that break the chorion and release the embryo (De Gaspar et al., 1999). Ethanol causes toxic effects to these enzyme activities, which could reduce or delay the hatching rate (Fraysse et al., 2006). Besides that, we observed that animals from Eth 1% group presented less frequency of spontaneous movement in the egg. Thus, we suggest that ethanol effects on hatching enzymes combined with its influence on embryonic activity within the egg may have led to hatching disruption. This result follows previous studies showing ethanol‐induced hatch and development delay starting at 1% concentration (Hallare et al., 2006).

Moreover, Ali et al. (2017) and Pelka et al. (2017) showed that some substances could modify the chorion structure, causing reshaping or shrinking, damaging the protective layer, and crashing the egg, releasing the animal at earlier stages. Another effect that should be considered is handling, as the early hatch caused by handling stress may affect animal development, leading to small body size or morphological alteration. However, all groups were exposed to handling, while only those exposed to ethanol of 0.5% and 1% showed hatching time changes that are more consistent with the drug treatment than handling stress.

Ethanol exposure effects were also evidenced by the abnormalities observed at 120 hpf (Figure 2). Ethanol 0.5% and 1% exposure led to comorbidities such as tail edema, yolk sac edema, and abnormal posture. Abnormal body formation would be expected due to the ethanol exposure at the pharyngula stage, a time in which the embryonic body axis straightens from its initial curvature about the yolk sac (Kimmel et al., 1995). Previous findings have suggested that alcohol exposure during development cause skeletal muscle malformation due to disruption of sonic hedgehog signaling, an important gene regulator for the somite formation, both in zebrafish and in mammals (Lombard et al., 2007; Nishimura et al., 2016; Zhang et al., 2014). This body malformation could also be related to lower spontaneous movement observed for both groups (0.5% and 1%), making it difficult for the embryo to get rid of the chorion and hatch.

Moreover, pericardial edema and the cardiac function's evaluation by heartbeat frequency reinforce the toxic effects of the low ethanol concentrations. While heartbeats were registered only until 104 hpf, because embryos after that point were wholly pigmented and counting was not trustable, differences between groups were observed (Figure 3). Embryos exposed to Eth 1% exhibited reduced heartbeat frequency, result in agreement with other authors' findings for ethanol‐treated animals (Bilotta et al., 2002; Dlugos & Rabin, 2010; Hallare et al., 2006). The heartbeat deficiency can incite heart edemas and vice versa, which was suggested to be related to the ethanol disruption in the central nervous system (CNS) or to alterations in particulars molecular pathways relates to the ethanol toxicity to genes related to cardiac formation and functioning (El‐Mas & Abdel‐Rahman, 2003; Hallare et al., 2006).

Another critical observation is alcohol's direct action on the developing brain. Rats exposed to ethanol at the same time points tested in this study showed structural changes in the brain, especially in the neurulation stage, which occurs early in pregnancy in humans (Fish et al., 2018). Here, we observed the effects of ethanol treatment on apoptosis levels during embryogenesis. Acridine orange is a nucleic acid‐selective fluorescent cationic dye, proposed as a rapid and inexpensive assay to investigate apoptotic damage in cells (Kim et al., 2014). The fluorescence intensity analyzed follows the same results found in morphologic observations, showing Eth 0.5% and Eth 1% groups with higher cellular death levels. This finding supports previous studies on ethanol apoptotic effects (Carvan et al., 2004; Cole & Ross, 2001). The drug's apoptogenic effects were shown to be related to the blockade of NMDA glutamate receptors and hyperactivation of GABA(A) receptors (Harris et al., 1995; Lovinger et al., 1989). The cell death caused during the nervous system formation may lead to several incorrect connections and reduced brain activity that ultimately affect behavior. Zebrafish treated with 0.25% ethanol—the lowest concentration used—showed apoptotic cells similar to the control group, suggesting 0.25% ethanol was not effective in causing damage to the embryo. This result was also observed for the other parameters evaluated (edemas, body size/structure, cardiac function, hatching). Although 0.25% ethanol seems not to cause any changes to the embryo as observed here and in a previous study regarding behavioral effects (Pinheiro‐da‐Silva et al., 2020), it should not be taken as a secure amount of ethanol. As shown by Baggio et al. (2020), even 0.25% ethanol provokes biochemical alterations that persist into adulthood. The nonobservable damage to brain development and consequent neurobehavioral disturbances are the most debilitating effects of ethanol on the fetus (Olney et al., 2001).

Finally, we showed that embryonic ethanol exposure leads to concentration‐dependent effects observable during development. These effects include parameters of the morphology, biochemistry, and behavior that change throughout ontogeny. Early ethanol exposure can cause minor and almost imperceptible alterations to gross morphology in zebrafish, which cannot be discarded as part of FASD. We reinforce that the zebrafish is a suitable and reliable model for FASD cases, suggesting that the threshold between FASD and FAS is very tenue and depends on the period and extension of exposure, as well as ethanol concentration and intrinsic characteristics of the individuals (genetics). Remarkably, even a single exposure to low concentrations of ethanol can cause several changes to the organism, possibly correlated with each other, and can be tracked to adulthood. It is essential to highlight that regarding embryonic ethanol exposure, there is no safe concentration to be used.

## CONFLICT OF INTEREST

The authors declare no conflict of interest.

## AUTHOR CONTRIBUTIONS

JPS conceived and performed the experiments. JPS and ACL designed the experiments, analyzed the data, and wrote the paper. Both authors contributed equally to the direction of this work.

### PEER REVIEW

The peer review history for this article is available at https://publons.com/publon/10.1002/brb3.2062.

## Supporting information

Supplementary Material

Supplementary Material

## Data Availability

The data that support the findings of this study are available from the corresponding author upon reasonable request.

## References

[brb32062-bib-0001] Ali, M. K. , Saber, S. P. , Taite, D. R. , Emadi, S. , & Irving, R. (2017). The protective layer of zebrafish embryo changes continuously with advancing age of embryo development (AGED). Journal of Toxicology and Pharmacology, 1(2), 009. 10.1021/tx300021

[brb32062-bib-0002] Arenzana, F. J. , Carvan, M. J. , Aijón, J. , Sánchez‐González, R. , Arévalo, R. , & Porteros, A. (2006). Teratogenic effects of ethanol exposure on zebrafish visual system development. Neurotoxicology and Teratology, 28(3), 342–348. 10.1016/j.ntt.2006.02.001 16574376

[brb32062-bib-0003] Baggio, S. , Mussulini, B. H. , de Oliveira, D. L. , Gerlai, R. , & Rico, E. P. (2018). Embryonic alcohol exposure leading to social avoidance and altered anxiety responses in adult zebrafish. Behavioural Brain Research, 352(August 2017), 62–69. 10.1016/j.bbr.2017.08.039 28882694

[brb32062-bib-0004] Baggio, S. , Zenki, K. , Martins Silva, A. , dos Santos, T. G. , Rech, G. , Lazzarotto, G. , & de Oliveira, D. L. (2020 Fetal alcohol spectrum disorders model alters the functionality of glutamatergic neurotransmission in adult zebrafish. NeuroToxicology, 78(December 2019), 152–160. 10.1016/j.neuro.2020.03.003 32173352

[brb32062-bib-0005] Bailey, J. M. , Oliveri, A. N. , & Levin, E. D. (2015). Pharmacological analyses of learning and memory in zebrafish (*Danio* *rerio*). Pharmacology Biochemistry and Behavior, 139, 103–111. 10.1016/j.pbb.2015.03.006 PMC4573775 25792292

[brb32062-bib-0006] Barr, H. M. , & Streissguth, A. P. (2001). Identifying maternal self‐reported alcohol use associated with fetal alcohol spectrum disorders. Alcoholism: Clinical and Experimental Research, 25(2), 283–287. 10.1111/j.1530-0277.2001.tb02210.x 11236844

[brb32062-bib-0007] Bilotta, J. , Barnett, J. A. , Hancock, L. , & Saszik, S. (2004). Ethanol exposure alters zebrafish development: A novel model of fetal alcohol syndrome. Neurotoxicology and Teratology, 26(6 SPEC., ISS.), 737–743. 10.1016/j.ntt.2004.06.011 15451038

[brb32062-bib-0008] Bilotta, J. , Saszik, S. , Givin, C. M. , Hardesty, H. R. , & Sutherland, S. E. (2002). Effects of embryonic exposure to ethanol on zebrafish visual function. Neurotoxicology and Teratology, 24(6), 759–766. 10.1016/S0892-0362(02)00319-7 12460658

[brb32062-bib-0009] Blader, P. , & Strähle, U. (1998). Ethanol impairs migration of the prechordal plate in the zebrafish embryo. Developmental Biology, 201(2), 185–201. 10.1006/dbio.1998.8995 9740658

[brb32062-bib-0010] Buske, C. , & Gerlai, R. (2011). Early embryonic ethanol exposure impairs shoaling and the dopaminergic and serotoninergic systems in adult zebrafish. Neurotoxicology and Teratology, 33(6), 698–707. 10.1016/j.ntt.2011.05.009 21658445 PMC3179771

[brb32062-bib-0011] Carvan, M. J. , Loucks, E. , Weber, D. N. , & Williams, F. E. (2004). Ethanol effects on the developing zebrafish: Neurobehavior and skeletal morphogenesis. Neurotoxicology and Teratology, 26(6), 757–768. 10.1016/j.ntt.2004.06.016 15451040

[brb32062-bib-0012] Chmielewski, C. E. , Hernandez, L. M. , Quesada, A. , Pozas, J. A. , Picabea, L. , & Prada, F. A. (1997). Effects of ethanol on the inner layers of chick retina during development. Alcohol, 14(4), 313–317. 10.1016/S0741-8329(97)87948-7 9209545

[brb32062-bib-0013] Cole, L. K. , & Ross, L. S. (2001). Apoptosis in the developing zebrafish embryo. Developmental Biology, 240(1), 123–142. 10.1006/dbio.2001.0432 11784051

[brb32062-bib-0014] De Gaspar, I. , Blanquez, M. J. , Fraile, B. , Paniagua, R. , & Arenas, M. I. (1999). The hatching gland cells of trout embryos: Characterisation of N‐and O‐linked oligosaccharides. Journal of Anatomy, 194(1), 109–118. 10.1046/j.1469-7580.1999.19410109.x 10227672 PMC1467899

[brb32062-bib-0015] Dlugos, C. A. , & Rabin, R. A. (2010). Structural and functional effects of developmental exposure to ethanol on the zebrafish heart. Alcoholism: Clinical and Experimental Research, 34(6), 1013–1021. 10.1111/j.1530-0277.2010.01176.x 20374213

[brb32062-bib-0016] El‐Mas, M. M. , & Abdel‐Rahman, A. A. (2003). Effects of chronic ethanol feeding on clonidine‐evoked reductions in blood pressure, heart rate, and their variability: Time‐domain analyses. Journal of Pharmacology and Experimental Therapeutics, 306(1), 271–278. 10.1124/jpet.102.048355 12660306

[brb32062-bib-0017] Fernandes, Y. , & Gerlai, R. (2009). Long‐term behavioral changes in response to early developmental exposure to ethanol in zebrafish. Alcoholism: Clinical and Experimental Research, 33(4), 601–609. 10.1111/j.1530-0277.2008.00874.x PMC2715552 19183139

[brb32062-bib-0018] Fernandes, Y. , Rampersad, M. , Jones, E. M. , & Eberhart, J. K. (2019). Social deficits following embryonic ethanol exposure arise in post‐larval zebrafish. Addiction Biology, 24(5), 898–907. 10.1111/adb.12649 30178621 PMC6629526

[brb32062-bib-0019] Fish, E. W. , Wieczorek, L. A. , Rumple, A. , Suttie, M. , Moy, S. S. , Hammond, P. , & Parnell, S. E. (2018). The enduring impact of neurulation stage alcohol exposure: A combined behavioral and structural neuroimaging study in adult male and female C57BL/6J mice. Behavioural Brain Research, 338(June 2017), 173–184. 10.1016/j.bbr.2017.10.020 29107713 PMC5726510

[brb32062-bib-0020] Fraysse, B. , Mons, R. , & Garric, J. (2006). Development of a zebrafish 4‐day embryo‐larval bioassay to assess toxicity of chemicals. Ecotoxicology and Environmental Safety, 63, 253–267. 10.1016/j.ecoenv.2004.10.015 16677909

[brb32062-bib-0021] Gil‐Mohapel, J. , Bianco, C. D. , Cesconetto, P. A. , Zamoner, A. , & Brocardo, P. S. (2019). Ethanol exposure during development, and brain oxidative stress. Neuroscience of alcohol. Elsevier Inc. 10.1016/b978-0-12-813125-1.00051-9

[brb32062-bib-0022] Grunwald, D. J. , & Eisen, J. S. (2002). Headwaters of the zebrafish—Emergence of a new model vertebrate. Nature Reviews Genetics, 3(9), 717–724. 10.1038/nrg892 12209146

[brb32062-bib-0023] Guerri, C. , Bazinet, A. , & Riley, E. P. (2009). Foetal alcohol spectrum disorders and alterations in brain and behaviour. Alcohol & Alcoholism, 44(2), 108–114. 10.1093/alcalc/agn105 19147799 PMC2724862

[brb32062-bib-0024] Hagan, J. F. , Balachova, T. , Bertrand, J. , Chasnoff, I. , Dang, E. , Fernandez‐Baca, D. , Kable, J. , Kosofsky, B. , Senturias, Y. N. , Singh, N. , Sloane, M. , Weitzman, C. , & Zubler, J. (2016). Neurobehavioral disorder associated with prenatal alcohol exposure. Pediatrics, 138(4), e20151553. 10.1542/peds.2015-1553 27677572 PMC5477054

[brb32062-bib-0025] Hallare, A. , Nagel, K. , Köhler, H. R. , & Triebskorn, R. (2006). Comparative embryotoxicity and proteotoxicity of three carrier solvents to zebrafish (*Danio* *rerio*) embryos. Ecotoxicology and Environmental Safety, 63(3), 378–388. 10.1016/j.ecoenv.2005.07.006 16125774

[brb32062-bib-0026] Harris, R. A. , Proctor, W. R. , McQuilkin, S. J. , Klein, R. L. , Mascia, M. P. , Whatley, V. , Whiting, P. J. , & Dunwiddie, T. V. (1995). Ethanol increases GABAA responses in cells stably transfected with receptor subunits. Alcoholism: Clinical and Experimental Research, 19(1), 226–232. 10.1111/j.1530-0277.1995.tb01496.x 7771653

[brb32062-bib-0027] Hashimoto, H. , Matsuo, Y. , Yokoyama, Y. , Toyohara, H. , & Sakaguchi, M. (1998). Induction of apoptosis in fish cells by hypertonic stress. Fisheries Science, 64(5), 820–825. 10.2331/fishsci.64.820

[brb32062-bib-0028] Hoyme, H. E. , May, P. A. , Kalberg, W. O. , Kodituwakku, P. , Gossage, J. P. , Trujillo, P. M. , Buckley, D. G. , Miller, J. H. , Aragon, A. S. , Khaole, N. , Viljoen, D. L. , Jones, K. L. , & Robinson, L. K. (2005). A practical clinical approach to diagnosis of fetal alcohol spectrum disorders: Clarification of the 1996 institute of medicine criteria. Pediatrics, 115(1), 39–47. 10.1542/peds.2004-0259 15629980 PMC1380311

[brb32062-bib-0029] Jones, K. , & Smith, D. (1973). Recognition of the fetal alcohol syndrome in early infancy. The Lancet, 302(7836), 999–1001. 10.1016/s0140-6736(73)91092-1 4127281

[brb32062-bib-0030] Jones, K. , Smith, D. , Ulleland, C. , & Streissguth, A. (1973). Pattern of malformation in offspring of chronic alcoholic mothers. The Lancet, 301(7815), 1267–1271. 10.1016/s0140-6736(73)91291-9 4126070

[brb32062-bib-0031] Kalueff, A. V. , Echevarria, D. J. , & Stewart, A. M. (2014). Gaining translational momentum: More zebrafish models for neuroscience research. Progress in Neuro‐Psychopharmacology and Biological Psychiatry, 55, 1–6. 10.1016/j.pnpbp.2014.01.022 24593944

[brb32062-bib-0032] Kim, E.‐A. , Lee, S.‐H. , Ko, C.‐I. , Cha, S.‐H. , Kang, M.‐C. , Kang, S.‐M. , Ko, S.‐C. , Lee, W.‐W. , Ko, J.‐Y. , Lee, J.‐H. , Kang, N. , Oh, J.‐Y. , Ahn, G. , Jee, Y. H. , & Jeon, Y.‐J. (2014). Protective effect of fucoidan against AAPH‐induced oxidative stress in zebrafish model. Carbohydrate Polymers, 102, 185–191. 10.1016/j.carbpol.2013.11.022 24507271

[brb32062-bib-0033] Kimmel, C. B. , Ballard, W. W. , Kimmel, S. R. , Ullmann, B. , & Schilling, T. F. (1995). Stages of embryonic development of the zebrafish. Developmental Dynamics, 203(3), 253–310. 10.1002/aja.1002030302 8589427

[brb32062-bib-0034] Kingdon, D. , Cardoso, C. , & McGrath, J. J. (2016). Research Review: Executive function deficits in fetal alcohol spectrum disorders and attention‐deficit/hyperactivity disorder – A meta‐analysis. Journal of Child Psychology and Psychiatry, 57(2), 116–131. 10.1111/jcpp.12451 26251262 PMC5760222

[brb32062-bib-0035] Lange, S. , Probst, C. , Gmel, G. , Rehm, J. , Burd, L. , & Popova, S. (2018). Global prevalence of fetal alcohol spectrum disorder among children and youth: A systematic review and meta‐analysis. Obstetrical & Gynecological Survey, 73(4), 189–191. 10.1097/01.ogx.0000532194.88210.00 PMC5710622 28828483

[brb32062-bib-0036] Lombard, Z. , Tiffin, N. , Hofmann, O. , Bajic, V. B. , Hide, W. , & Ramsay, M. (2007). Computational selection and prioritization of candidate genes for fetal alcohol syndrome. BMC Genomics, 8(1), 389. 10.1186/1471-2164-8-389 17961254 PMC2194724

[brb32062-bib-0037] Lovinger, D. M. , White, G. , & Weight, F. F. (1989). Ethanol inhibits NMDA‐activated ion current in hippocampal neurons. Science, 243(4899), 1721–1724. 2467382 10.1126/science.2467382

[brb32062-bib-0038] Marrs, J. A. , Clendenon, S. G. , Ratcliffe, D. R. , Fielding, S. M. , Liu, Q. , & Bosron, W. F. (2010). Zebrafish fetal alcohol syndrome model: Effects of ethanol are rescued by retinoic acid supplement. Alcohol, 44(7–8), 707–715. 10.1016/j.alcohol.2009.03.004 20036484 PMC2889201

[brb32062-bib-0039] Matsui, J. I. , Egana, A. L. , Sponholtz, T. R. , Adolph, A. R. , & Dowling, J. E. (2006). Effects of ethanol on photoreceptors and visual function in developing zebrafish. Investigative Ophthalmology and Visual Science, 47(10), 4589–4597. 10.1167/iovs.05-0971 17003456 PMC2408731

[brb32062-bib-0040] May, P. A. , Baete, A. , Russo, J. , Elliott, A. J. , Blankenship, J. , Kalberg, W. O. , Buckley, D. , Brooks, M. , Hasken, J. , Abdul‐Rahman, O. , Adam, M. P. , Robinson, L. K. , Manning, M. , & Hoyme, H. E. (2014). Prevalence and characteristics of fetal alcohol spectrum disorders. Pediatrics, 134(5), 855–866. 10.1542/peds.2013-3319 25349310 PMC4210790

[brb32062-bib-0041] Muralidharan, P. , Sarmah, S. , & Marrs, J. A. (2015). Zebrafish retinal defects induced by ethanol exposure are rescued by retinoic acid and folic acid supplement. Alcohol, 49(2), 149–163. 10.1016/j.alcohol.2014.11.001 25541501 PMC4339401

[brb32062-bib-0042] Nishimura, Y. , Inoue, A. , Sasagawa, S. , Koiwa, J. , Kawaguchi, K. , Kawase, R. , Maruyama, T. , Kim, S. , & Tanaka, T. (2016). Using zebrafish in systems toxicology for developmental toxicity testing. Congenital Anomalies, 56(1), 18–27. 10.1111/cga.12142 26537640

[brb32062-bib-0043] Olney, J. W. , Wozniak, D. F. , Jevtovic‐Todorovic, V. , & Ikonomidou, C. (2001). Glutamate signaling and the fetal alcohol syndrome. Mental Retardation and Developmental Disabilities Research Reviews, 7(4), 267–275. 10.1002/mrdd.1037 11754521

[brb32062-bib-0044] Pan, Y. , Chatterjee, D. , & Gerlai, R. (2012). Strain dependent gene expression and neurochemical levels in the brain of zebrafish: Focus on a few alcohol related targets. Physiology and Behavior, 107(5), 773–780. 10.1016/j.physbeh.2012.01.017 22313674 PMC3368073

[brb32062-bib-0045] Pelka, K. E. , Henn, K. , Keck, A. , Sapel, B. , & Braunbeck, T. (2017). Size does matter – Determination of the critical molecular size for the uptake of chemicals across the chorion of zebrafish (*Danio* *rerio*) embryos. Aquatic Toxicology, 185, 1–10. 10.1016/j.aquatox.2016.12.015 28142078

[brb32062-bib-0046] Pinheiro‐da‐Silva, J. , Agues‐Barbosa, T. , & Luchiari, A. C. (2020). Embryonic exposure to ethanol increases anxiety‐like behavior in fry zebrafish. Alcohol and Alcoholism, 55(6), 581–590. 10.1093/alcalc/agaa087 32886092

[brb32062-bib-0047] Roozen, S. , Peters, G. J. Y. , Kok, G. , Townend, D. , Nijhuis, J. , & Curfs, L. (2016). Worldwide prevalence of fetal alcohol spectrum disorders: A systematic literature review including meta‐analysis. Alcoholism: Clinical and Experimental Research, 40(1), 18–32. 10.1111/acer.12939 26727519

[brb32062-bib-0048] Sampson, P. D. , Streissguth, A. P. , Bookstein, F. L. , Little, R. E. , Clarren, S. K. , Dehaene, P. , Hanson, J. W. , & Graham, J. M. (1997). Incidence of fetal alcohol syndrome and prevalence of alcohol‐related neurodevelopmental disorder. Teratology, 56(5), 317–326. 10.1002/(SICI)1096-9926(199711)56:5<317:AID-TERA5>3.0.CO;2-U 9451756

[brb32062-bib-0049] Shan, S. D. , Boutin, S. , Ferdous, J. , & Ali, D. W. (2015). Ethanol exposure during gastrulation alters neuronal morphology and behavior in zebrafish. Neurotoxicology and Teratology, 48, 18–27. 10.1016/j.ntt.2015.01.004 25599605

[brb32062-bib-0050] Stewart, A. , Braubach, O. , Spitsbergen, J. , Gerlai, R. , & Kalueff, A. V. (2014). Zebrafish models for translational neuroscience research: From tank to bedside. Trends in Neurosciences, 37(5), 264–278. 10.1016/j.tins.2014.02.011 24726051 PMC4039217

[brb32062-bib-0051] Streissguth, A. P. (1997). Fetal alcohol syndrome: A guide for families and communities. Paul H Brookes Publishing.

[brb32062-bib-0052] Sylvain, N. J. , Brewster, D. L. , & Ali, D. W. (2010). Zebrafish embryos exposed to alcohol undergo abnormal development of motor neurons and muscle fibers. Neurotoxicology and Teratology, 32(4), 472–480. 10.1016/j.ntt.2010.03.001 20211721

[brb32062-bib-0053] Tenkova, T. , Young, C. , Dikranian, K. , Labruyere, J. , & Olney, J. W. (2003). Ethanol‐induced apoptosis in the developing visual system during synaptogenesis. Investigative Ophthalmology & Visual Science, 44(7), 2809–2817. 10.1167/iovs.02-0982 12824217

[brb32062-bib-0054] Terasaki, L. S. , Gomez, J. , & Schwarz, J. M. (2016). An examination of sex differences in the effects of early‐life opiate and alcohol exposure. Philosophical Transactions of the Royal Society B: Biological Sciences, 371(1688), 20150123. 10.1098/rstb.2015.0123 PMC4785906 26833841

[brb32062-bib-0055] Yamashita, M. (2003). Apoptosis in zebrafish development. Comparative Biochemistry and Physiology Part B: Biochemistry and Molecular Biology, 136(4), 731–742. 10.1016/j.cbpc.2003.08.013 14662298

[brb32062-bib-0056] Zhang, C. , Frazier, J. M. , Chen, H. , Liu, Y. , Lee, J.‐A. , & Cole, G. J. (2014). Molecular and morphological changes in zebrafish following transient ethanol exposure during defined developmental stages. Neurotoxicology and Teratology, 44, 70–80. 10.1016/j.ntt.2014.06.001 24929233 PMC4120066

