# Supplementary figures and images for: Embryonic ethanol exposure on zebrafish early development
Source: Brain Behav. 2021 May 3;11(6):e02062. doi: 10.1002/brb3.2062 (PMC8213935; doi:10.1002/brb3.2062)

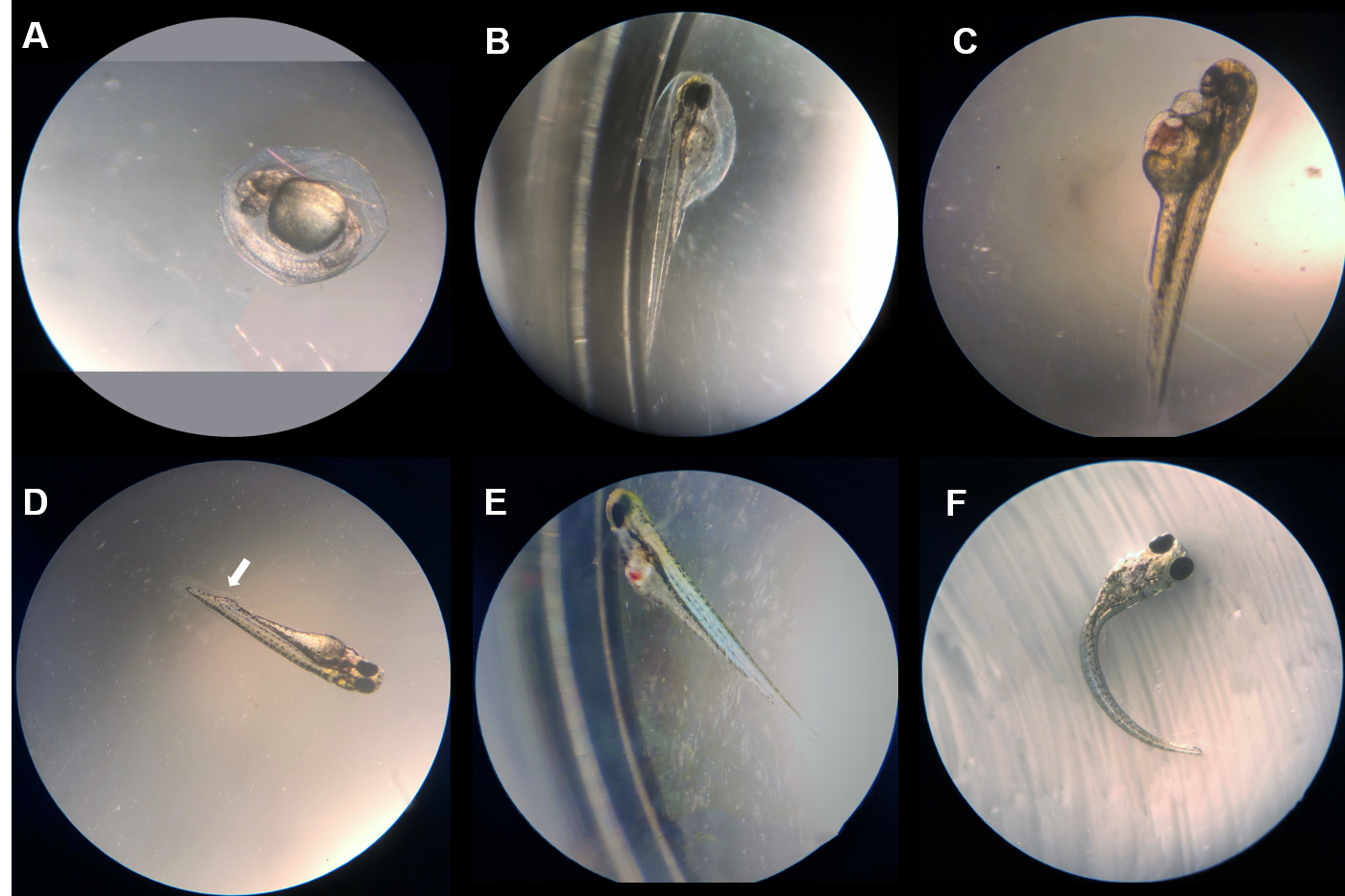

Supplement: Supplementary file 1 — Supplementary Material [file BRB3-11-e02062-s002.png]
