# Supplementary material for: Embryonic ethanol exposure on zebrafish early development
Source: Brain Behav. 2021 May 3;11(6):e02062. doi: 10.1002/brb3.2062 (PMC8213935; doi:10.1002/brb3.2062)
Supplement: Supplementary file 2 — Supplementary Material [file BRB3-11-e02062-s001.docx]

**Supporting Information 1:** Morphological abnormalities observed during zebrafish embryo development after ethanol exposure. a) Shrunken chorion after ethanol exposure; b) Larvae hatched with head in the eggshell; c) yolk sac anomaly; d) tail edema, e) heart edema f) larvae full developed with bend posture.
